# Autism and Intellectual Disability Are Differentially Related to Sociodemographic Background at Birth

**DOI:** 10.1371/journal.pone.0017875

**Published:** 2011-03-30

**Authors:** Helen Leonard, Emma Glasson, Natasha Nassar, Andrew Whitehouse, Ami Bebbington, Jenny Bourke, Peter Jacoby, Glenys Dixon, Eva Malacova, Carol Bower, Fiona Stanley

**Affiliations:** 1 Telethon Institute for Child Health Research, Centre for Child Health Research, The University of Western Australia, West Perth, Western Australia, Australia; 2 School of Population Health, The University of Western Australia, Perth, Western Australia, Australia; 3 Perinatal Research, Kolling Institute of Medical Research, University of Sydney, Sydney, New South Wales, Australia; 4 National Drug Research Institute, Curtin University, Perth, Western Australia, Australia; The University of Queensland, Australia

## Abstract

**Background:**

Research findings investigating the sociodemographics of autism spectrum disorder (ASD) have been inconsistent and rarely considered the presence of intellectual disability (ID).

**Methods:**

We used population data on Western Australian singletons born from 1984 to 1999 (n = 398,353) to examine the sociodemographic characteristics of children diagnosed with ASD with or without ID, or ID without ASD compared with non-affected children.

**Results:**

The profiles for the four categories examined, mild-moderate ID, severe ID, ASD without ID and ASD with ID varied considerably and we often identified a gradient effect where the risk factors for mild-moderate ID and ASD without ID were at opposite extremes while those for ASD with ID were intermediary. This was demonstrated clearly with increased odds of ASD without ID amongst older mothers aged 35 years and over (odds ratio (OR) = 1.69 [CI: 1.18, 2.43]), first born infants (OR = 2.78; [CI: 1.67, 4.54]), male infants (OR = 6.57 [CI: 4.87, 8.87]) and increasing socioeconomic advantage. In contrast, mild-moderate ID was associated with younger mothers aged less than 20 years (OR = 1.88 [CI: 1.57, 2.25]), paternal age greater than 40 years (OR = 1.59 [CI: 1.36, 1.86]), Australian-born and Aboriginal mothers (OR = 1.60 [CI: 1.41, 1.82]), increasing birth order and increasing social disadvantage (OR = 2.56 [CI: 2.27, 2.97]). Mothers of infants residing in regional or remote areas had consistently lower risk of ASD or ID and may be linked to reduced access to services or under-ascertainment rather than a protective effect of location.

**Conclusions:**

The different risk profiles observed between groups may be related to aetiological differences or ascertainment factors or both. Untangling these pathways is challenging but an urgent public health priority in view of the supposed autism epidemic.

## Introduction

A number of sociodemographic factors have been associated with autism spectrum disorders (ASD) with and without intellectual disability (ID), including social class and ethnicity. In studies undertaken in the US [1], [2] but not in Denmark [3], higher social class was found to be associated with ASD, particularly ASD without ID [1], [2]. Ethnic and racial disparities in ASD have also been predominantly limited to the US [1], [4], [5]. In an earlier Western Australian study which included children with ID with and without ASD but not children with ASD without ID, we found that Aboriginality was inversely associated with ASD [6]. The impact of migration has previously been demonstrated mainly in Nordic countries with an increase in ASD in children whose mothers were born outside the study area [7]–[9]. However, the effect of maternal country of birth on diagnosis of ASD with or without ID has not been examined. The relationship between ASD and other sociodemographic factors, such as parental age, birth order and residential location (urban/rural) also remains unclear with results inconsistent across studies [10]–[22].

We have access to population data relating to the diagnosis of ASD and ID in Western Australian children [23]. Using the Western Australian Data Linkage System (WADLS) [24], we are also able to link these data to other population-based sources such as the Midwives Notification System and the Register of Births, which provide information about the antenatal and perinatal details of pregnancies and births as well as the sociodemographic characteristics of the parents. By only releasing deidentified data to researchers, the Western Australian Data Linkage System can conserve the privacy of individual patients. In contrast to consent-based research, data linkage systems such as this provide the advantage of conserving the privacy of all patients regardless of whether or not they would have given consent to the use of information [24].

To test the hypothesis that there are differences in distribution of sociodemographic factors of children with ASD and children with ID we used data linkage to examine these factors in affected children and to compare them with non-affected children. In particular, we investigated level of economic resources, parental age, ethnicity, maternal country of birth, marital status and maternal height as well as birth order and infant gender.

## Methods

### Ethics statement

Ethical approval for the study was provided by the University of Western Australia and the Confidentiality of Health Information Committee for the Department of Health, WA.

### Study population and data sources

The study population comprised all children born in Western Australia (WA) between 1st January, 1984 and 31st December, 1999 and alive in 2005 (n = 393,329). Children with a primary diagnosis of ASD or ID or both (ASD with ID) formed the case group. As done previously [23], the WA Data Linkage System was used to combine individual-level data on all births in WA with ASD and ID data sources. The resulting dataset was de-identified prior to being provided to the researchers such that no consent was required in relation to the study population [24].

The population-based ascertainment of children with ASD in WA including the data sources, diagnostic procedures and service provision arrangements over this time period has previously been reported in considerable detail [23]. In brief this involved the establishment of a Central Diagnostic Panel in 1991 and the introduction of cross-disciplinary reporting protocols in 1997 such that diagnosis could be made in a standardised and rigorous way [23]. The primary diagnostic tool used up to the mid-1990s was the DSM-IIIR [25] and in 1994 this was superseded by the DSM-IV criteria [26] and in 2000 by the DSM-IV-TR [27]. Children with ASD were identified from three overlapping sources: 1) the Disability Services Commission of WA database (the government agency that is the primary service provider and assessment agency for children with ASD and ID); 2) the WA Register of Autism Spectrum Disorders, a prospective surveillance system of newly diagnosed cases since 1999; and 3) a retrospective dataset based on a comprehensive audit and individual case note review of all ASD cases born in WA between 1984 and 1995 and diagnosed by 1999 [23]. Children with ID were identified from the Intellectual Disability Exploring Answers Database, a WA population-based register of children with ID [28]. ID diagnostic codes have been assigned by physicians using the American Association on Mental Retardation classification system [29] and, as done previously [6], [30], categorised as biomedical or otherwise. By excluding 895 cases with a biomedical cause for the ID we, like others [31], [32], were able to separate out isolated or unspecified ID from what has been described as co-developmental or biopathological ID.

The analysis was limited to singleton births to allow comparison with other recent key studies which either focussed on singletons [16], [19], [33] or adjusted for multiplicity [20]. Of the 1179 singleton children identified with an ASD, 826 (70.1%) were classified as childhood autism (aka Autistic disorder), 217 (18.4%) as Pervasive Developmental Disorder not otherwise specified (PDD-NOS), 64 (5.4%) as Asperger syndrome and in 72 (6.1%) the classification was not specified into an ASD subcategory. In 452 (38.5%) of the children, there was no identified ID (ASD without ID). In the remainder (n = 727), ID had been identified or, at least, not excluded (ASD with ID). A total of 4576 children were diagnosed with an ID with no biomedical cause and with no ASD, of whom 237 (5.2%) had severe and 4315 (91%) mild-moderate ID. In 183 (3.8%) children, the ID level was unspecified and these were included in the mild-moderate category.

We report here on the sociodemographic characteristics of these 1179 singleton cases of ASD (categorised according to association with ID) and the 4576 singleton cases of ID without ASD (categorised by level of ID) compared with the remainder of the singleton children born in WA between 1984 and 1999 and alive in 2005 and not identified as having ID or ASD (n = 376,529).

Infant characteristics examined were sex and birth order. Parental characteristics examined were maternal ethnicity, country of birth and height, marital status and maternal and paternal age at birth. Mother's country of birth was assigned using the Standard Australian Classification of Countries 1998 Revision 2.03.14 [34]. The Accessibility/Remoteness Index of Australia (ARIA), based on the mother's postcode of residence at time of infant's birth, was used as an indicator of geographical remoteness [35] to assign cases to one of three categories: major cities, inner regional, or outer regional and remote. We also used the index of economic resources (SEIFA, a neighbourhood level summary measure for each census Collection District (approximately 200 dwellings) from the Australian Bureau of Statistics 1996 Census of Population and Housing [36]) grouped into sextiles, as a proxy for mother's socioeconomic status at the time of the child's birth. Other categories were compared with the least resourced sextile. As intrauterine growth restriction is a known risk factor for ID [37], we adjusted for this in the analysis using percentage of optimal birth weight (POBW) [38]. POBW is the ratio of the observed birth weight to optimal birth weight taking into account sex, birthweight, gestational age and maternal height [38]. It was categorized into seven groups <75, 75–<85, 85–<95, 95–<105, 105–<115, 115–<125, and ≥125 percent, with the middle group, 95–<105 percent, used as the referent in the analysis. The percentage of missing data was 0.05% for marital status, 0.28% for POBW, 0.79% for maternal country of birth, 1.47% for maternal height, 1.58% for ARIA, 5.52% for paternal age and 10.44% for index of economic resources.

Stata [39] was used for statistical analysis. Proportions by each level of categorical variables for cases with mild-moderate ID, severe ID, ASD with ID, and ASD without ID were compared with the population group of children without ID or ASD. The association between sociodemographic characteristics and each diagnostic grouping was assessed using multivariable analysis to take into account potential confounding by explanatory factors using complete case analysis. Maternal country of birth was omitted from multivariable analyses because of small cell sizes. Adjustment for birth year as a categorical variable was included in all models. Odds ratios and 95% confidence intervals were calculated separately for each case group using multinomial logistic regression.

## Results

### Univariate analysis (see Tables S1, S2, S3, S4)

**Figure 1:**
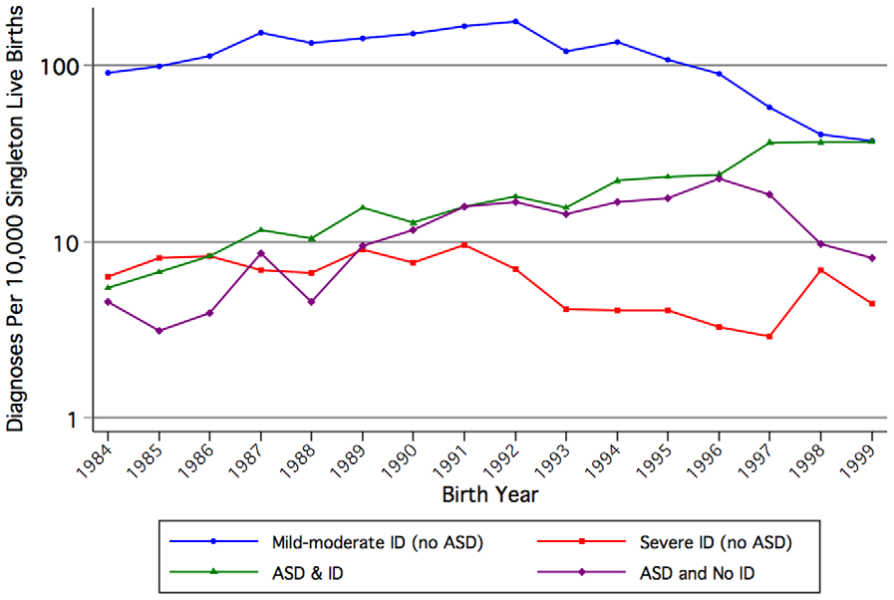
Prevalence of Autism Spectrum Disorder (ASD) or/and Intellectual Disability (ID) of unknown cause among infants born 1984 to 1999, per 10,000 singleton live births.

The birth prevalence of ASD (with and without ID) in singleton births increased over time from 10 per 10,000 births in 1984 to 45 per 10,000 in 1999, peaking at 55 per 10,000 in 1997 (Figure 1). The sharpest increase over time was seen for ASD with ID whilst less acute in recent years for ASD without ID. The prevalence of mild-moderate ID increased in the early years but subsequently declined. The pattern for severe ID was more stable with a peak in 1991 and a gradual decrease over time.

**Figure 2:**
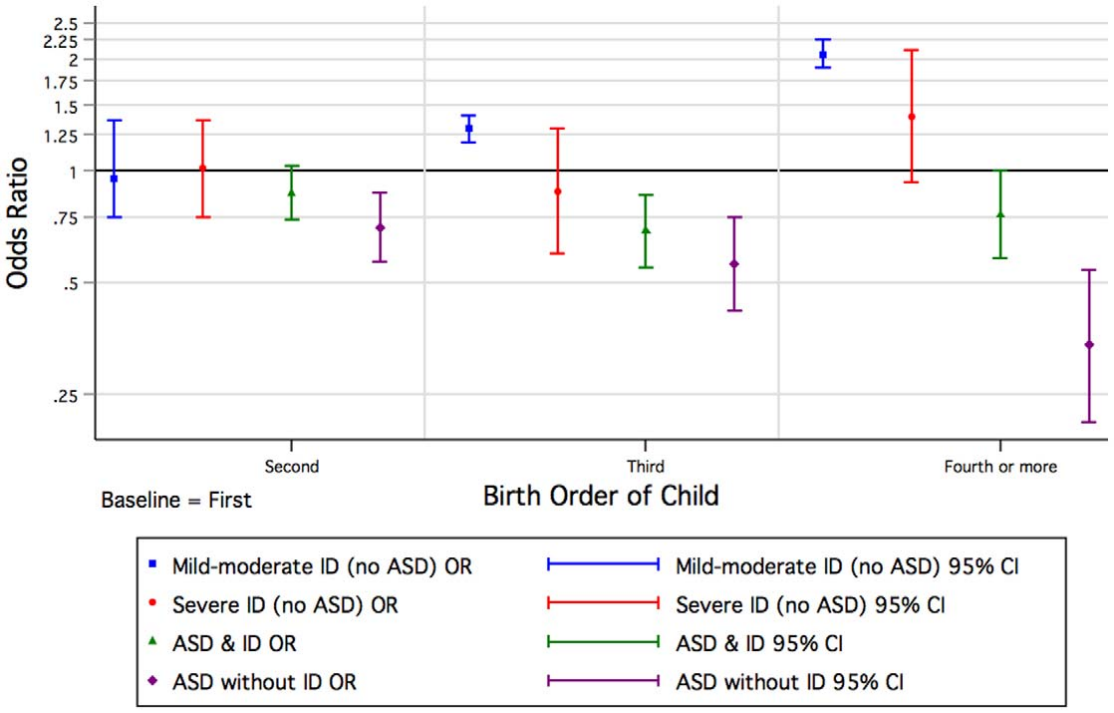
Odds of Autism Spectrum Disorder (ASD) or/and Intellectual Disability (ID) of unknown cause by birth order of child (univariate) born 1984 to 1999.

Males had an increased likelihood of diagnosis of ASD without ID (OR = 6.63 [CI: 5.02, 8.75]) and the odds remained elevated, but decreased with presence of ID (OR = 4.48 [CI: 3.70, 5.42]) (Table S1). The odds were still increased but to a lesser extent for children with mild-moderate ID. When compared with those who were first born, the odds for ASD with and without ID were reduced for those second or later born. In contrast the odds for children with mild-moderate and severe ID were increased for later born children (Figure 2, Table S1).

**Figure 3:**
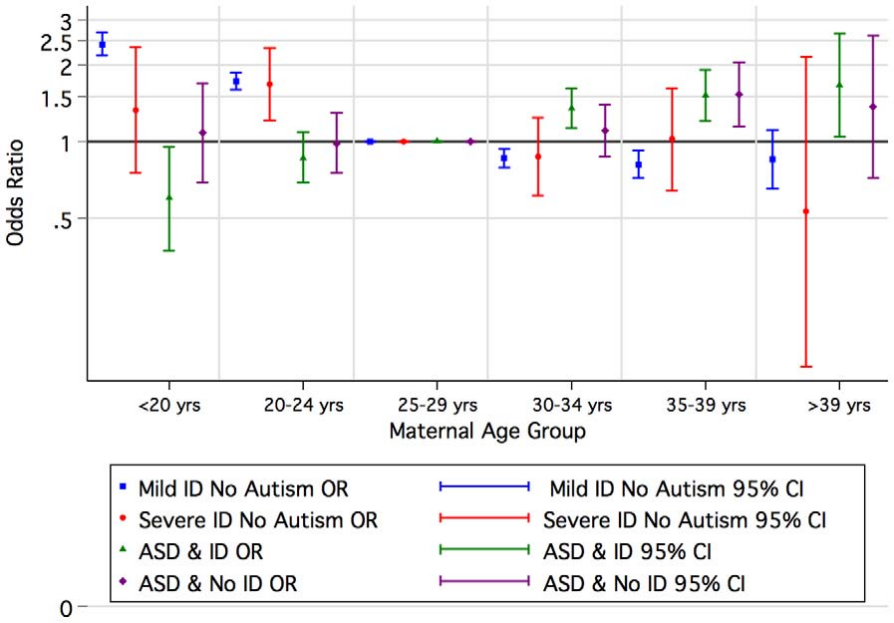
Odds of Autism Spectrum Disorder (ASD) or/and Intellectual Disability (ID) of unknown cause by maternal agegroup by birth year, 1984–1999 (univariate).

**Figure 4:**
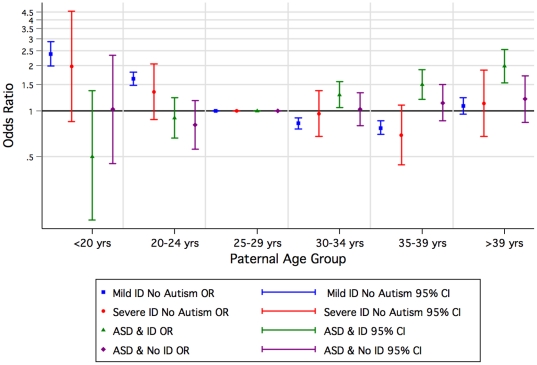
Odds of Autism Spectrum Disorder (ASD) or/and Intellectual Disability (ID) of unknown cause by paternal agegroup by birth year, 1984–1999 (univariate).

Compared with children of mothers aged 25–29 years, children of older mothers had an increased risk of ASD with and without ID in contrast to the pattern for mild-moderate and severe ID where the risk increased for younger mothers (Figure 3, Table S2). A similar pattern was observed for older fathers where the increased risk was greatest for ASD with ID (Figure 4, Table S2).

Compared with married or *defacto* married women, single and separated, widowed or divorced women had an increased odds of having a child with mild-moderate or severe ID (Table S2), whilst the effect was reversed for ASD particularly for those with ID. The mean maternal height ranged from 162.2 cm (95% CI 162.0–162.4) for those with mild-moderate ID and 162.1 cm (95% CI 161.2-163.0) for those with severe ID to 163.7 cm (95% CI 163.2–164.3) for those with ASD and ID and 164.3 cm (95% CI 163.7–164.9) for those with ASD without ID. For those with no ID or ASD, the mean maternal height was 163.4 (95% CI 163.4 –163.4).

Compared with Caucasian mothers, the odds of having a child with ASD were reduced for Aboriginal mothers and increased for Asian mothers but for having a child with mild-moderate ID the effects were reversed (Table S3). Mothers born in South East Asia and North East Asia were more likely than mothers born in Australia (or the rest of Oceania) to have a child with ASD and ID (Table 1).

**Table 1:** Maternal birthplace by diagnosis of Autism Spectrum Disorder (ASD) or/and Intellectual Disability (ID) of unknown cause.

Maternal Birth Place	Not ID	Mild-moderate ID	OR 95% CI	Severe ID	OR 95% CI	ASD + ID	OR 95% CI	ASD without ID	OR 95% CI
1 Oceania and Antarctica	278,569	3,498	1	177	1	508	1	327	1
	(73.98%)	(80.62%)		(74.63%)		(69.19%)		(72.35%)	
2 North West Europe	54,522	500	0.73	28	0.81	104	1.06	78	1.22
	(14.48%)	(11.52%)	(0.66–0.80)	(11.81%)	(0.54–1.20)	(14.31%)	(0.86–1.30)	(17.26%)	(0.95–1.56)
3 Southern and Eastern Europe	8,187	77	0.75	8	1.54	23	1.56	5	0.52
	(2.17%)	(1.77%)	(0.60–0.94)	(3.38%)	(0.76–3.12)	(3.16%)	(1.02–2.36)	(1.11%)	(0.22–1.26)
4 North Africa and Middle East	1,832	16	0.70	3	2.58	7	2.12	0	
	(0.49%)	(0.37%)	(0.42–1.14)	(1.27%)	(0.82–8.08)	(0.96%)	(1.00–4.47)	(0%)	
5 South East Asia	15,629	100	0.51	10	1.01	46	1.63	18	0.98
	(4.15%)	(2.30%)	(0.42–0.62)	(4.22%)	(0.53–1.90)	(6.33%)	(1.20–2.20)	(3.98%)	(0.61–1.58)
6 North East Asia	2,615	12	0.37	2	1.20	13	2.75	5	1.63
	(0.69%)	(0.28%)	(0.21–0.64)	(0.84%)	(0.30–4.85)	(1.79%)	(1.59–4.78)	(1.11%)	(0.67–3.94)
7 South and Central Asia	4956	31	0.50	2	0.64	9	1.01	8	1.38
	(1.32%)	(0.71%)	(0.35–.71)	(0.84%)	(0.16–2.56)	(1.24%)	(0.52–1.94)	(1.77%)	(0.68–2.77)
8 Americas	2,237	11	0.39	1	0.70	4	1.24	4	1.52
	(0.59%)	(0.25%)	(0.22–0.71)	(0.42%)	(0.10–5.02)	(0.88%)	(0.51–3.00)	(0.46%)	(0.57–4.08)
9 Sub-Saharan Africa	5,028	35	0.55	3	0.94	15	1.65	6	1.02
	(1.34%)	(0.81%)	(0.40–0.77)	(1.27%)	(0.30–2.94)	(2.06%)	(0.99–2.76)	(1.33%)	(0.45–2.28)
missing	2,954	59		3		2		1	

**Figure 5:**
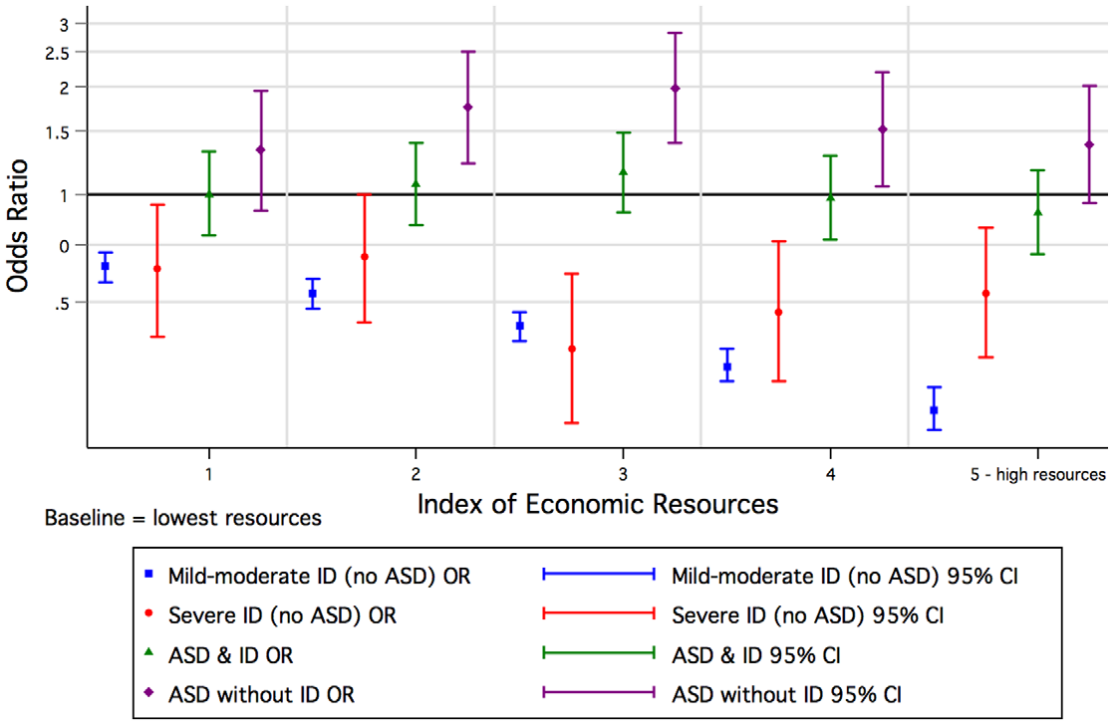
Odds of Autism Spectrum Disorder (ASD) or/and Intellectual Disability (ID) of unknown cause by index of economic resources, relating to mother's residence at the time of the child's birth, 1984–1999 (univariate).

Mothers living in regional or remote areas were less likely to have a child diagnosed with ASD without ID compared with mothers residing in metropolitan areas (Table S3). For mild-moderate ID, the odds were slightly increased for children of mothers living in an outer regional or remote area, but, once Aboriginal children were excluded, the effect was reversed (OR = 0.90 [CI: 0.83, 0.98]). Compared with the most poorly economically resourced group there was an increased odds of ASD without ID with increasing levels of socioeconomic status, although the relationship was not linear (Figure 5, Table S4). In contrast, a declining social gradient was associated with increased risk of mild-moderate ID.

### Multivariable analysis

For mild-moderate ID, most relationships seen in the univariate analysis persisted in the multivariable analysis with an increased likelihood associated with infants of male gender and higher birth order and mothers who were younger, Aboriginal, economically disadvantaged and small statured (Table S5). The one relationship that changed was that for those whose mothers were not living in the metropolitan area there was now a reduced odds of a diagnosis of mild-moderate ID. With severe ID, where numbers were much smaller, the relationship with younger maternal age (20–24 years) still persisted as did the relationship with maternal stature and some of the relationships with economic disadvantage.

For analyses where ASD with ID (Table S5) was the outcome, the variables that remained strong predictors were increasing birth year, infant sex, birth order, ethnicity and paternal age greater than 39 years. For ASD without ID as the outcome (Table S5) the explanatory factors were again later birth year, male sex, first births and increasing socioeconomic status. However in contrast to ASD with ID, there was also an increased risk of ASD without ID for offspring of mothers aged 35–39 years (OR = 1.69 [CI: 1.18, 2.43]). As with mild-moderate ID (p<0.001), living in a regional or remote area reduced the odds of a diagnosis of ASD without ID (p = 0.05).

## Discussion

This is the first population-based study of ASD to examine comprehensively the association of a range of sociodemographic effects according to the presence or absence of ID and provide comparisons with children diagnosed either solely with ID or without ASD or ID. Most previous research has investigated ASD as a single entity and, despite inconsistent findings, has rarely evaluated whether the presence of ID might modify any association. This is important given recent concerns that there may be some diagnostic accretion to ASD from ID and a potential change in the characteristics of ASD children over time [23], [40]–[42].

Because of some already known and contrasting attributes (eg birth order) associated with ID of unknown cause [31], [43] and ASD [10], [12], [33], we postulated a gradient effect in which the risk factors for mild-moderate ID and ASD without ID were at the extremes and those for ASD with ID intermediary. Indeed these were the striking patterns we saw with infant sex, birth order (Figure 2) and maternal height (which could reflect socioeconomic status) [44], [45]. Aboriginality also followed the pattern with an increased risk for mild-moderate and severe ID converting to an extreme protective effect for ASD without ID. In contrast for children of Asian mothers, the risk of ASD was higher with comorbid ID than for ASD without ID, whilst the effect for mild-moderate but not severe ID was protective. Socioeconomic status as characterised by the index of economic resources provided a similar but less dramatic picture. For older mothers, we found an increased risk for ASD without ID, and a similar but attenuated (once adjusted for paternal age and other factors) effect for ASD with ID. In contrast, younger mothers had an increased risk for mild-moderate ID. However, advanced paternal age was associated most with ASD with accompanying ID.

A major strength of this study is our access to population-based data on ASD and ID and the ability to compare risk factors for these two outcomes. We nevertheless acknowledge limitations such as the difficulty in adequately assessing cognitive function in children with ASD. We therefore conservatively restricted our group of children with ASD without ID to those who, based on assessment information, we were confident did not have an IQ deficit. Any misclassification of this group would have meant that we may have underestimated some of the differences we identified. Only children born between 1984 and 1999 were included to allow the maximum follow-up for diagnosis until the time at which the linked dataset was made available. We also adjusted for birth year in the multivariable analysis to take account of any variation across birth cohorts. As have Californian studies in particular [16], [19], [33], we restricted our analysis to singletons. However we recognise that, despite being population-based, the number of births each year in Western Australia represents less than 5% of that of California. Therefore the power of our analysis and ability for further stratification (such as for parental age groups within restricted subsets of the other parent's age as undertaken on Californian data [19]) was limited. Also small cell sizes prevented us from taking account of maternal country of birth in the multivariate analysis. Further, we acknowledge that the area-based measure of socioeconomic status remains a surrogate for an individual measure and that we had no unit data on maternal education or income.

An early Californian study found the same pattern as we did of decreasing sex ratio with increasing presence of ID but did not examine the effect of co-morbid ID on other sociodemographic variables [46]. Two other studies, both from the US [1], [2], have subsequently examined a range of sociodemographic factors in ASD and accounted for ID. However, we know of none other than this and our own previous study [6] which have compared the sociodemographic characteristics of ASD and ID in a population cohort. Findings from the Atlanta study [1], consistent with ours, highlight that a greater proportion of children with ASD without ID were from higher socioeconomic backgrounds and were more likely to be male than children with ASD and ID. Using data from the Autism and Developmental Disabilities Monitoring Network, the most recent of the US studies [2] identified a SES gradient but only in ASD without ID. Moreover the gradient was stronger in children with a pre-existing diagnosis than in those identified through the surveillance program. The SES gradients we detected demonstrated considerable contrast between ID where the prevalence decreased with increasing socioeconomic advantage and ASD without ID where there was some increase but to a much lesser extent and not in a dose-response manner as seen in the 2010 US study [2]. This could relate to the more equitable health system we have in Australia compared with the US, although not all Western Australian children are diagnosed with ASD within the public system [47].

Offspring of younger mothers have been reported to be at risk of mild-moderate ID [48] and offspring of older mothers at risk of ASD [10], [12], [16], [33], [46], [49]. However, as we found in our study for ASD with ID (but not for ASD without ID), once adjusted for other factors, the maternal age effect for ASD may become less evident [3], [8], [12], [15], [22], [33]. We also found that increasing paternal age was associated with an increased risk of ASD with ID (but not of ASD without ID) even when adjusted for other factors. In a recent study of a 10 year Californian birth cohort, the risk of ASD was found to increase with advancing maternal age irrespective of paternal age but the paternal age effect was most evident when the mother was aged under 30 years [19]. In another recent Californian study, King et al. suggested that pooling data across birth cohorts could inflate the risk associated with paternal age and, after undertaking a “decomposition” strategy to separate parental age effects, they concluded that advanced maternal age posed a greater risk than advanced paternal age [21]. They also highlighted the changes that may have occurred in the characteristics of children with ASD over time including the proportion with associated ID. The most recently published study to investigate parental age in autism used multiple study designs including a Swedish birth cohort, a family-based study and a meta-analysis [22]. Contrary to the two recent Californian studies [19], [21] which had much larger case numbers of autism, no maternal age effect was identified in the Swedish birth cohort analysis. However, the meta-analysis which included an earlier Californian study but neither of the two recent ones [19], [21], confirmed the paternal age effect identified in the birth cohort study. Despite the extensive literature ours appears to be the first study to separately examine the parental age effect associated with ASD with and without accompanying ID. The inability to do this was noted as a shortcoming in the recent Swedish cohort analysis [22]. We found that increasing paternal age was associated with an increased risk of ASD with ID (but not of ASD without ID) even when adjusted for other factors, whilst for maternal age the pattern of association was reversed. It has been suggested that different mechanisms may be responsible for paternally and maternally mediated age effects [19]. Therefore our subtle but important findings, not able to be elucidated in previous research [8], [12], [15], [16], [22], [33], illustrate the importance of taking into account the presence of co-morbid ID when investigating underlying causal pathways in ASD.

Having a non-Australian born mother generally provided a protective effect for mild-moderate ID whilst the risk of ASD with ID increased slightly for children of mothers from North East and South East Asia, North Africa and the Middle East. These results are consistent with recent UK findings where marked immigration effects were identified particularly for mothers from the Caribbean, Africa and Asia [50]. Most previous studies examining this effect had been based in Scandinavia [7]–[9]. More recent studies have investigated particularly the apparent increase in ASD in children born to Somali mothers in Sweden and postulated a possible causal link with Vitamin D deficiency [51], [52]. An additional cluster of Somali children has also been reported in the US state of Minnesota [53], [54]. There have been few investigations of ASD in Aboriginal populations but an increased risk of ASD, particularly with ID, has been reported for African-American children [1]. However in a separate study, it was found that even if African-American children, particularly with comorbid ID, met the ASD case definition, it was not necessarily recorded in their case notes [5]. Hispanic children in Texas have also been found to be less likely to be diagnosed with autism [4]. Furthermore, minority children were also underrepresented in a Dutch ASD assessment centre, although this bias disappeared when paediatricians were specifically invited in a scenario situation to assess children for ASD [55]. Thus there is evidence of ASD underascertainment in minority groups and this could partly account for our findings in relation to the Australian Aboriginal population.

It is generally acknowledged that there is considerable interplay among the various social, environmental and biological factors affecting the occurrence of autism. The sociodemographic gradients we identified in relation to ASD and ID may relate to ascertainment or aetiology or both. It is likely that access to ASD diagnostic services has been inequitable across Western Australia and children in rural areas may have been disadvantaged [23]. The process for ASD diagnosis is more complex than that for ID and Aboriginal children may be more readily assigned an ID than an ASD label. As we have previously described for Western Australia [56], there have been major sociocultural influences over the past few decades which have led to the destigmatisation of autism such that it has become a much more socially acceptable diagnosis generally accruing more benefits for the child (and family) than if the diagnosis was only ID. Thus parents are playing a greater role in seeking and securing an ASD diagnosis for their children, especially when, as in Western Australia, there are financial incentives to do so [23]. Similar scenarios are also occurring across the developed world and diagnostic substitution is acknowledged to play a determining role in the increased prevalence of autism [41], [57]. A recent Californian study has estimated that social influence and information diffusion can account for as great a proportion of the increase as maternal age, socioeconomic status and genetic determinants [58]. This was demonstrated by showing that children living close to a child with autism are more likely to be diagnosed with autism than ID [58]. If statistical power were adequate, replicating the Californian spatial study in Western Australia might explain the mechanisms underlying the decline in prevalence of mild-moderate ID and increase in ASD with ID over time as well as the low prevalence of ASD in Aboriginal children.

Diagnostic dynamics and social diffusion aside, given the high heritability index, it is assumed that a high proportion of ASD cases result from a combination of genetic susceptibility and environmental exposure rather than from a single cause or mutated gene [59]. A number of hypothetical models have been proposed involving methylation, the immune system, neurotoxic exposure to heavy metals, oxidative stress, folic acid and Vitamin D to explain how the interaction of environmental and genetic factors might be responsible for this increased prevalence of autism [60]. What is of concern is that the demonstrated models are not specific for autism and might just as likely produce any neurological phenotype. It is essential, as we [23] and others [41] have done, to examine in parallel the trajectories of ID without ASD as well as ASD with and without ID. It is equally important to examine over time the composition of ASD in terms of the contribution of co-morbid ID.

Despite the evidence for heritability and the research resources invested in this exploration to date, the exact genetic basis for autism and how this could contribute to increased prevalence over time remains elusive. Unlike other genetic mechanisms, rates of denovo germ-line mutations (DNMs) can be susceptible to rapid social/environmental change. Although it has been postulated that heritability may have been overstated in the past it has been possible to demonstrate the contribution of DNMs to the aetiology of autism by investigating the concordance amongst twin pairs over time [42]. Thus sociodemographic change-namely an increase in parental age- appears to be influencing the genetic contribution to autism [42]. In keeping with this finding a molecular genetic study has recently demonstrated an excess of potentially deleterious DNMs in ASD as well as schizophrenia [61]. Another study has postulated a digenic model, with one gene (G1) responsible for the ID symptoms and another one (G2) responsible for ASD symptoms again involving DNMs [62]. The second gene could be associated with particular personality traits in G1 non-mutated relatives without ID. Our findings of a paternal age effect specifically in ASD with ID would be particularly consistent with this theory. In linking together both environmental and genetic factors, Kinney and colleagues [53] suggest that many of the environmental factors included in the models presented by Currenti (2010) [60] could well be acting through the generation of DNMs.

We suggest that the different risk profiles we have observed for maternal and paternal age for ASD with ID and ASD without ID represent aetiological differences for the two groups. Alternatively, as demonstrated best by sex ratio and birth order, the group with ASD with ID may represent a dilution of the pure ASD phenotype (i.e. ASD without ID). In either of these scenarios, we should caution about the repercussions for analysis and interpretation of combining such groups in epidemiological and genetic investigations.

In summary, we have used population-based disability and perinatal registers to comprehensively compare sociodemographic factors of ASD and ID. The profiles of the observed associations were often found to be complementary. The mechanisms underlying these relationships involve either ascertainment related factors or aetiological determinants or a combination of both. In the light of the supposed autism epidemic and the secular changes occurring in parental age at childbirth, untangling these pathways is vital, challenging and an urgent public health priority.

## Supporting Information

Table S1 Infant characteristics by diagnosis of Intellectual Disability (ID) of unknown cause and Autism Spectrum Disorder (ASD) with and without ID.
(DOC)

Table S2 Intellectual Disability (ID) of unknown cause and Autism Spectrum Disorder (ASD) with and without ID by maternal agegroup, paternal agegroup and marital status at the time of the child's birth.
(DOC)

Table S3 Intellectual Disability (ID) of unknown cause and Autism Spectrum Disorder (ASD) with and without ID by maternal ethnicity and area of residence at the time of the child's birth.
(DOC)

Table S4 Socioeconomic indices based on mother's residence at time of infant's birth for Intellectual Disability (ID) of unknown cause and Autism Spectrum Disorder (ASD) with and without ID.
(DOC)

Table S5 Multivariate analysis of the associations of sociodemographic factors and diagnosis of intellectual disability (ID) of unknown cause and autism spectrum disorder (ASD) with and without ID.
(DOC)
