# Supplementary material for: Autism and Intellectual Disability Are Differentially Related to Sociodemographic Background at Birth
Source: PLoS One. 2011 Mar 30;6(3):e17875. doi: 10.1371/journal.pone.0017875 (PMC3068153; doi:10.1371/journal.pone.0017875)
Supplement: Table S1 — Infant characteristics by diagnosis of Intellectual Disability (ID) of unknown cause and Autism Spectrum Disorder (ASD) with and without ID. (DOC) [file pone.0017875.s001.doc]

Table S1 Infant characteristics by diagnosis of Intellectual Disability (ID) of unknown cause and Autism Spectrum Disorder (ASD) with and without ID

| Category | Not ID | Mild-moderate ID | OR(95% CI) | Severe ID | OR(95% CI) | ASD + ID | OR(95% CI) | ASD without ID | OR(95% CI) |
| --- | --- | --- | --- | --- | --- | --- | --- | --- | --- |
| Gender of child |  |  |  |  |  |  |  |  |  |
| Female | 184,085(48.89%) | 1,616(37.24%) | 1 | 85(35.86%) | 1 | 128(17.61%) | 1 | 57(12.61%) | 1 |
| Male | 192,444(51.11%) | 2,723(62.76%) | 1.61(1.52 - 1.71) | 152(64.14%) | 1.71(1.31 - 2.23) | 599(82.39%) | 4.48(3.70 - 5.42) | 398(87.39%) | 6.63(5.02 - 8.75) |
| Birth order |  |  |  |  |  |  |  |  |  |
| 1^st^ born | 149,701(39.76%) | 1,518(34.99%) | 1 | 92(38.82%) | 1 | 328(45.12%) | 1 | 236(52.21%) | 1 |
| 2^nd^ born | 126,462(33.59%) | 1,223(28.19%) | 0.95(0.75 - 1.37) | 79(33.33%) | 1.02(0.75 - 1.37) | 242(33.29%) | 0.87(0.74 - 1.03) | 140(30.97%) | 0.70(0.57 - 0.87) |
| 3^rd^ born | 64,455(17.12%) | 848(19.54%) | 1.30(1.19 - 1.41) | 35(14.77%) | 0.88(0.60 - 1.30) | 97(13.34%) | 0.69(0.55 - 0.86) | 57(12.61%) | 0.56(0.42 - 0.75) |
| 4^th^ or later born | 35,909(9.54%) | 750(17.29%) | 2.06(1.89 - 2.25) | 31(13.08%) | 1.40(0.93 - 2.11) | 60(8.25%) | 0.76(0.58 - 1.00) | 19(4.2%) | 0.34(0.21 - 0.54) |
| missing | 2 | 0 |  | 0 |  | 0 |  | 0 |  |
