# Supplementary material for: Autism and Intellectual Disability Are Differentially Related to Sociodemographic Background at Birth
Source: PLoS One. 2011 Mar 30;6(3):e17875. doi: 10.1371/journal.pone.0017875 (PMC3068153; doi:10.1371/journal.pone.0017875)
Supplement: Table S2 — Intellectual Disability (ID) of unknown cause and Autism Spectrum Disorder (ASD) with and without ID by maternal agegroup, paternal agegroup and marital status at the time of the child's birth. (DOC) [file pone.0017875.s002.doc]

Table S2 Intellectual Disability (ID) of unknown cause and Autism Spectrum Disorder (ASD) with and without ID by maternal agegroup, paternal agegroup and marital status at the time of the child’s birth

| Category | Not ID | Mild-moderate ID | OR(95% CI) | Severe ID | OR(95% CI) | ASD + ID | OR(95% CI) | ASD without ID | OR(95% CI) |
| --- | --- | --- | --- | --- | --- | --- | --- | --- | --- |
| Maternal age group | | | | | | | | | |
| <20 years | 18,523(4.92%) | 449(10.35%) | 2.41(2.17 - 2.69) | 14(5.91%) | 1.33(0.75 - 2.35) | 19(2.61%) | 0.60(0.37 - 0.95) | 22(4.87%) | 1.08(0.69 - 1.69) |
| 20-24 years | 73,164(19.43%) | 1,267(29.2%) | 1.72(1.6 - 1.86) | 70(29.54%) | 1.68(1.21 - 2.33) | 109(14.99%) | 0.86(0.69 - 1.09) | 79(17.48%) | 0.98(0.75 - 1.29) |
| 25-29 years | 131,740(34.99%) | 1,323(30.49%) | 1 | 75(31.65%) | 1 | 227(31.22%) | 1 | 145(32.08%) | 1 |
| 30-34 years | 106,655(28.33%) | 918(21.16%) | 0.86(0.79 - 0.93) | 53(22.36%) | 0.87(0.61 - 1.24) | 249(34.25%) | 1.35(1.13 - 1.62) | 129(28.54%) | 1.10(0.87 - 1.39) |
| 35-39 years | 39,801(10.57%) | 325(7.49%) | 0.81(0.72 - 0.92) | 23(9.7%) | 1.02(0.64 - 1.62) | 104(14.31%) | 1.52(1.2 - 1.91) | 67(14.82%) | 1.53(1.14 - 2.04) |
| >39 years | 6,645(1.76%) | 57(1.31%) | 0.85(0.65 - 1.11) | 2(0.84%) | 0.53(0.13 - 2.15) | 19(2.61%) | 1.66(1.04 - 2.65) | 10(2.21%) | 1.37(0.72 - 2.60) |
| missing | 1 | 0 | - | 0 | - | 0 | - | 0 | - |
| Paternal age group | | | | | | | | | |
| <20 years | 5,087(1.35%) | 127(2.93%) | 2.38(1.98 - 2.87) | 6(2.53%) | 1.97(0.85 - 4.56) | 4(0.55%) | 0.5(0.19 - 1.36) | 6(1.33%) | 1.03(0.45 - 2.33) |
| 20-24 years | 39,852(10.58%) | 681(15.69%) | 1.63(1.48 - 1.8) | 32(13.5%) | 1.34(0.88 - 2.05) | 56(7.7%) | 0.9(0.66 - 1.22) | 37(8.19%) | 0.81(0.56 - 1.17) |
| 25-29 years | 103,546(27.5%) | 1,084(24.98%) | 1 | 62(26.16%) | 1 | 162(22.28%) | 1 | 119(26.33%) | 1 |
| 30-34 years | 114,958(30.53%) | 997(22.98%) | 0.83(0.76 - 0.9) | 66(27.85%) | 0.96(0.68 - 1.36) | 230(31.64%) | 1.28(1.05 - 1.56) | 136(30.09%) | 1.03(0.8 - 1.32) |
| 35-39 years | 62,928(16.71%) | 510(11.75%) | 0.77(0.7 - 0.86) | 26(10.97%) | 0.69(0.44 - 1.09) | 147(20.22%) | 1.49(1.19 - 1.87) | 82(18.14%) | 1.13(0.86 - 1.5) |
| >39 years | 29,759(7.9%) | 336(7.74%) | 1.08(0.95 - 1.22) | 20(8.44%) | 1.12(0.68 - 1.86) | 92(12.65%) | 1.98(1.53 - 2.55) | 41(9.07%) | 1.2(0.84 - 1.71) |
| missing | 20,399(5.42%) | 604(13.92%) |  | 25(10.55%) |  | 36(4.95%) |  | 31(6.86%) |  |
| Marital status (of mothers) | | | | | | | | | |
| Married or Defacto | 336,424(89.35%) | 3,348(77.16%) | 1 | 192(81.01%) | 1 | 662(91.06%) | 1 | 405(89.6%) | 1 |
| Never married/Single | 35,823(9.51%) | 883(20.35%) | 2.48(2.3 - 2.67) | 40(16.88%) | 1.96(1.39 - 2.75) | 52(7.15%) | 0.74(0.56 - 0.98) | 42(9.29%) | 0.97(0.71 - 1.34) |
| Widowed, divorced or separated | 4,103(1.09%) | 106(2.44%) | 2.6(2.13 - 3.16) | 5(2.11%) | 2.14(0.88 - 5.19) | 13(1.79%) | 1.61(0.93 - 2.79) | 5(1.11%) | 1.01(0.42 - 2.45) |
| missing | 179 | 2 | - | 0 | - | 0 | - | 0 | - |
