# Supplementary material for: Autism and Intellectual Disability Are Differentially Related to Sociodemographic Background at Birth
Source: PLoS One. 2011 Mar 30;6(3):e17875. doi: 10.1371/journal.pone.0017875 (PMC3068153; doi:10.1371/journal.pone.0017875)
Supplement: Table S3 — Intellectual Disability (ID) of unknown cause and Autism Spectrum Disorder (ASD) with and without ID by maternal ethnicity and area of residence at the time of the child's birth. (DOC) [file pone.0017875.s003.doc]

Table S3 Intellectual Disability (ID) of unknown cause and Autism Spectrum Disorder (ASD) with and without ID

by maternal ethnicity and area of residence at the time of the child’s birth

| Category | Not ID | Mild-moderate ID | OR(95% CI) | Severe ID | OR(95% CI) | ASD + ID | OR(95% CI) | ASD without ID | OR(95% CI) |
| --- | --- | --- | --- | --- | --- | --- | --- | --- | --- |
| Maternal ethnicity |  |  |  |  |  |  |  |  |  |
| Caucasian | 329,926(87.62%) | 3,449(79.49%) | 1 | 195(82.28%) | 1 | 640(88.03%) | 1 | 422(93.36%) | 1 |
| Aboriginal | 20,775(5.52%) | 709(16.34%) | 3.26(3.01 - 3.54) | 22(9.28%) | 1.79(1.15 - 2.78) | 8(1.10%) | 0.20(0.10 - 0.40) | 2(0.44%) | 0.08 |
| Asian | 19,097(5.07%) | 121(2.79%) | 0.61(0.51 - 0.73) | 14(5.91%) | 1.24(0.72 - 2.13) | 57(7.84%) | 1.54(1.17 - 2.02) | 26(5.75%) | 1.06(0.72 - 1.58) |
| Other | 6,731(1.79%) | 60(1.38%) | 0.85(0.66 - 1.10) | 6(2.53%) | 1.51(0.67 - 3.40) | 22(3.03%) | 1.68(1.10 - 2.58) | 2(0.44%) | 0.23(0.06 - 0.93) |
| Index of Remoteness* |  |  |  |  |  |  |  |  |  |
| Major Cities | 243,721(64.73%) | 2,745(63.26%) | 1 | 157(66.24%) | 1 | 522(71.80%) | 1 | 336(74.34%) | 1 |
| Inner Regional | 38,204(10.15%) | 451(10.39%) | 1.05(0.95 - 1.16) | 24(10.13%) | 0.98(0.63 - 1.50) | 57(7.84%) | 0.7(0.53 - 0.92) | 40(8.85%) | 0.76(0.55 - 1.05) |
| Outer Regional and Remote | 88,614(23.53%) | 1,107(25.51%) | 1.11(1.03 - 1.19) | 50(21.10%) | 0.88(0.64 - 1.20) | 134(18.43%) | 0.71(0.58 - 0.85) | 71(15.71%) | 0.58(0.45 - 0.75) |
| missing | 5,990 | 36 |  | 6 |  | 14 |  | 5 |  |
