# Supplementary material for: Autism and Intellectual Disability Are Differentially Related to Sociodemographic Background at Birth
Source: PLoS One. 2011 Mar 30;6(3):e17875. doi: 10.1371/journal.pone.0017875 (PMC3068153; doi:10.1371/journal.pone.0017875)
Supplement: Table S4 — Socioeconomic indices based on mother's residence at time of infant's birth for Intellectual Disability (ID) of unknown cause and Autism Spectrum Disorder (ASD) with and without ID. (DOC) [file pone.0017875.s004.doc]

Table S4 Socioeconomic indices based on mother’s residence at time of infant’s birth for Intellectual Disability (ID) of unknown cause and Autism Spectrum Disorder (ASD) with and without ID

| Category | Not ID | Mild-moderate ID | OR(95% CI) | Severe ID | OR(95% CI) | ASD + ID | OR(95% CI) | ASD without ID | OR(95% CI) |
| --- | --- | --- | --- | --- | --- | --- | --- | --- | --- |
| Index of economic resources^a^ |  |  |  |  |  |  |  |  |  |
| 0 | 54,858(14.57%) | 1,209(27.86%) | 1 | 58(24.47%) | 1 | 106(14.58%) | 1 | 46(10.18%) | 1 |
| 1 | 53,826(14.3%) | 743(17.12%) | 0.63(0.57 - 0.69) | 35(14.77%) | 0.62(0.4 - 0.94) | 105(14.44%) | 1.01(0.77 - 1.32) | 60(13.27%) | 1.33 |
| 2 | 55,279(14.68%) | 645(14.87%) | 0.53(0.48 - 0.58) | 39(16.46%) | 0.67(0.44 - 1) | 114(15.68%) | 1.07(0.82 - 1.39) | 81(17.92%) | 1.75(1.22 - 2.51) |
| 3 | 56,563(15.02%) | 534(12.31%) | 0.43(0.39 - 0.47) | 22(9.28%) | 0.37(0.23 - 0.6) | 126(17.33%) | 1.15(0.89 - 1.49) | 94(20.8%) | 1.98(1.39 - 2.82) |
| 4 | 57,918(15.38%) | 422(9.73%) | 0.33(0.3 - 0.37) | 29(12.24%) | 0.47(0.3 - 0.74) | 110(15.13%) | 0.98(0.75 - 1.28) | 74(16.37%) | 1.52(1.05 - 2.2) |
| 5 | 58,731(15.6%) | 329(7.58%) | 0.25(0.22 - 0.29) | 33(13.92%) | 0.53(0.35 - 0.81) | 101(13.89%) | 0.89(0.68 - 1.17) | 68(15.04%) | 1.38(0.95 - 2.01) |
| missing | 39,354 | 457 |  | 21 |  | 65 |  | 29 |  |

^a^ 0= few resources, 5 = high resources etc.
