# Supplementary material for: Autism and Intellectual Disability Are Differentially Related to Sociodemographic Background at Birth
Source: PLoS One. 2011 Mar 30;6(3):e17875. doi: 10.1371/journal.pone.0017875 (PMC3068153; doi:10.1371/journal.pone.0017875)
Supplement: Table S5 — Multivariate analysis of the associations of sociodemographic factors and diagnosis of intellectual disability (ID) of unknown cause and autism spectrum disorder (ASD) with and without ID. (DOC) [file pone.0017875.s005.doc]

Table S5 Multivariate analysis of the associations of sociodemographic factors and diagnosis of intellectual disability (ID) of unknown cause

and autism spectrum disorder (ASD) with and without ID

|  | Category | Mild-moderate ID |  | Severe ID |  | ASD + ID |  | ASD without ID |  |
| --- | --- | --- | --- | --- | --- | --- | --- | --- | --- |
|  |  | OR | 95% CI | OR | 95% CI | OR | 95% CI | OR | 95% CI |
| Sex | Female | baseline |  | baseline |  | baseline |  | baseline |  |
|  | Male | 1.64 | (1.53 - 1.76) | 1.72 | (1.28 - 2.31) | 4.51 | (3.66 - 5.56) | 6.57 | (4.87 - 8.87) |
| Birth order | 1st born | baseline |  | baseline |  | baseline |  | baseline |  |
|  | 2nd born | 1.17 | (1.07 - 1.28) | 1.2 | (0.85 - 1.69) | 0.83 | (0.69 - 0.99) | 0.67 | (0.53 - 0.84) |
|  | 3rd born | 1.62 | (1.46 - 1.8) | 1.1 | (0.71 - 1.72) | 0.67 | (0.52 - 0.86) | 0.59 | (0.43 - 0.8) |
|  | 4th or more | 2.55 | (2.27 - 2.87) | 1.45 | (0.85 - 2.45) | 0.64 | (0.46 - 0.89) | 0.36 | (0.22 - 0.6) |
| Maternal age group | <20 years | 1.88 | (1.57 - 2.25) | 0.91 | (0.37 - 2.26) | 0.73 | (0.38 - 1.42) | 0.96 | (0.46 - 2.01) |
|  | 20-24 years | 1.51 | (1.36 - 1.67) | 1.54 | (1.01 - 2.33) | 0.88 | (0.66 - 1.17) | 0.87 | (0.62 - 1.23) |
|  | 25-29 years | baseline |  | baseline |  | baseline |  | baseline |  |
|  | 30-34 years | 0.79 | (0.72 - 0.88) | 1.01 | (0.67 - 1.52) | 1.11 | (0.9 - 1.38) | 1.08 | (0.82 - 1.42) |
|  | 35-39 years | 0.66 | (0.57 - 0.77) | 1 | (0.55 - 1.84) | 1.16 | (0.86 - 1.54) | 1.69 | (1.18 - 2.43) |
|  | >39 years | 0.5 | (0.36 - 0.69) | 0.56 | (0.13 - 2.47) | 1.3 | (0.77 - 2.21) | 1.61 | (0.77 - 3.34) |
| Paternal age group | <20 years | 1.18 | (0.93 - 1.49) | 1.42 | (0.48 - 4.16) | 0.72 | (0.24 - 2.15) | 1.2 | (0.42 - 3.44) |
|  | 20-24 years | 1.1 | (0.98 - 1.24) | 1.05 | (0.65 - 1.69) | 0.93 | (0.64 - 1.33) | 0.84 | (0.54 - 1.31) |
|  | 25-29 years | baseline |  | baseline |  | baseline |  | baseline |  |
|  | 30-34 years | 1.05 | (0.95 - 1.16) | 1.05 | (0.7 - 1.55) | 1.12 | (0.89 - 1.41) | 0.91 | (0.59 - 1.19) |
|  | 35-39 years | 1.1 | (0.97 - 1.25) | 0.74 | (0.42 - 1.28) | 1.26 | (0.96 - 1.65) | 0.84 | (0.53 - 1.29) |
|  | >39 years | 1.59 | (1.36 - 1.86) | 1.22 | (0.65 - 2.31) | 1.44 | (1.04 - 2) | 0.83 | (0.83 - 1.93) |
| Ethnicity | Caucasian | baseline |  | baseline |  | baseline |  | baseline |  |
|  | Aboriginal | 1.6 | (1.41 - 1.82) | 1.42 | (0.77 - 2.61) | 0.38 | (0.17 - 0.86) | 0 |  |
|  | Asian | 0.59 | (0.49 - 0.72) | 1.13 | (0.62 - 2.06) | 1.24 | (0.91 - 1.68) | 1.03 | (0.67 - 1.57) |
|  | Other | 0.75 | (0.57 - 0.99) | 1.31 | (0.53 - 3.2) | 1.43 | (0.89 - 2.3) | 0.27 | (0.07 - 1.09) |
| Index of economic resources | 0 | baseline |  | baseline |  | baseline |  | baseline |  |
|  | 1 | 0.77 | (0.7 - 0.85) | 0.76 | (0.48 - 1.2) | 0.84 | (0.63 - 1.13) | 1.27 | (0.83 - 1.93) |
|  | 2 | 0.68 | (0.61 - 0.75) | 0.81 | (0.51 - 1.27) | 0.86 | (0.65 - 1.13) | 1.43 | (0.96 - 2.14) |
|  | 3 | 0.57 | (0.51 - 0.64) | 0.52 | (0.31 - 0.88) | 0.87 | (0.66 - 1.15) | 1.54 | (1.04 - 2.28) |
|  | 4 | 0.46 | (0.4 - 0.52) | 0.61 | (0.37 - 1) | 0.74 | (0.56 - 0.99) | 1.24 | (0.83 - 1.86) |
|  | 5 | 0.39 | (0.34 - 0.44) | 0.71 | (0.43 - 1.15) | 0.71 | (0.53 - 0.95) | 1.18 | (0.78 - 1.79) |
| Index of remoteness | Major Cities | baseline |  | baseline |  | baseline |  | baseline |  |
|  | Inner Regional | 0.87 | (0.78 - 0.98) | 0.94 | (0.58 - 1.53) | 0.76 | (0.56 - 1.03) | 0.68 | (0.46 - 1.01) |
|  | Outer Regional and Remote | 0.73 | (0.67 - 0.81) | 0.8 | (0.54 - 1.19) | 0.88 | (0.7 - 1.1) | 0.74 | (0.54 - 1) |
| Marital status | Married-Defacto | baseline |  | baseline |  | baseline |  | baseline |  |
|  | Single | 1.31 | (1.17 - 1.48) | 1.25 | (0.74 - 2.09) | 0.82 | (0.55 - 1.23) | 0.68 | (0.4 - 1.14) |
|  | Widowed, divorced or separated | 1.65 | (1.29 - 2.12) | 1.38 | (0.44 - 4.38) | 1.44 | (0.68 - 3.05) | 1.88 | (0.77 - 4.59) |
| Maternal Height |  | 0.98 | (0.98 - 0.99) | 0.98 | (0.96 - 1) | 1 | (0.99 - 1.02) | 1.01 | (0.99 - 1.03) |
| POBW | normal | baseline |  | baseline |  | baseline |  | baseline |  |
|  | 85 - <95 | 1.25 | (1.14 - 1.37) | 1.25 | (0.84 - 1.85) | 1.12 | (0.91 - 1.38) | 1.14 | (0.88 - 1.48) |
|  | 75 - <85 | 1.91 | (1.71 - 2.12) | 1.85 | (1.16 - 2.94) | 1.13 | (0.84 - 1.52) | 0.96 | (0.65 - 1.43) |
|  | <75 | 2.85 | (2.43 - 3.34) | 6.22 | (3.75 - 10.33) | 2.02 | (1.31 - 3.13) | 1.29 | (0.65 - 2.54) |
|  | 105 - <115 | 0.97 | (0.87 - 1.09) | 0.99 | (0.62 - 1.56) | 1.05 | (0.84 - 1.32) | 1.06 | (0.8 - 1.41) |
|  | 115 - <125 | 1.12 | (0.96 - 1.31) | 0.83 | (0.39 - 1.75) | 0.89 | (0.62 - 1.28) | 1.25 | (0.84 - 1.85) |
|  | >125 | 1.38 | (1.09 - 1.74) | 1.9 | (0.81 - 4.44) | 1.2 | (0.71 - 2.03) |  |  |
| Birth year | 1984 | baseline |  | baseline |  | baseline |  | baseline |  |
|  | 1985 | 1.04 | (0.83 - 1.31) | 1.3 | (0.59 - 2.82) | 1.37 | (0.52 - 3.61) | 0.49 | (0.15 - 1.62) |
|  | 1986 | 1.23 | (0.99 - 1.52) | 1.32 | (0.61 - 2.85) | 1.94 | (0.79 - 4.75) | 0.69 | (0.24 - 1.98) |
|  | 1987 | 1.75 | (1.43 - 2.14) | 1.07 | (0.48 - 2.39) | 3.05 | (1.31 - 7.08) | 2.16 | (0.94 - 4.93) |
|  | 1988 | 1.51 | (1.23 - 1.86) | 1.16 | (0.52 - 2.55) | 2.72 | (1.16 - 6.37) | 1.1 | (0.43 - 2.79) |
|  | 1989 | 1.58 | (1.29 - 1.94) | 1.48 | (0.7 - 3.15) | 4.05 | (1.79 - 9.14) | 2.34 | (1.04 - 5.28) |
|  | 1990 | 1.56 | (1.28 - 1.91) | 1.19 | (0.55 - 2.61) | 2.85 | (1.23 - 6.59) | 2.24 | (1 - 5.04) |
|  | 1991 | 1.89 | (1.55 - 2.3) | 1.5 | (0.7 - 3.18) | 4.13 | (1.83 - 9.31) | 3.19 | (1.46 - 6.97) |
|  | 1992 | 1.97 | (1.61 - 2.39) | 1.07 | (0.48 - 2.41) | 3.63 | (1.6 - 8.24) | 3.69 | (1.71 - 7.96) |
|  | 1993 | 1.27 | (1.03 - 1.57) | 0.57 | (0.22 - 1.48) | 3.66 | (1.61 - 8.28) | 3.21 | (1.47 - 6.99) |
|  | 1994 | 1.53 | (1.24 - 1.88) | 0.66 | (0.26 - 1.65) | 5.01 | (2.26 - 11.14) | 3.79 | (1.76 - 8.14) |
|  | 1995 | 1.23 | (0.99 - 1.53) | 0.83 | (0.35 - 1.96) | 5.35 | (2.42 - 11.84) | 3.61 | (1.67 - 7.77) |
|  | 1996 | 1.08 | (0.87 - 1.35) | 0.58 | (0.22 - 1.5) | 5.38 | (2.44 - 11.9) | 4.6 | (2.17 - 9.75) |
|  | 1997 | 0.7 | (0.55 - 0.9) | 0.52 | (0.19 - 1.41) | 8.94 | (4.12 - 19.41) | 3.79 | (1.76 - 8.15) |
|  | 1998 | 0.5 | (0.38 - 0.66) | 1.34 | (0.61 - 2.93) | 7.92 | (3.63 - 17.25) | 1.88 | (0.82 - 4.3) |
|  | 1999 | 0.45 | (0.34 - 0.6) | 0.61 | (0.24 - 1.59) | 8.66 | (3.98 - 18.82) | 1.63 | (0.7 - 3.83) |
